# Whole Blood Transcriptomics Is Relevant to Identify Molecular Changes in Response to Genetic Selection for Feed Efficiency and Nutritional Status in the Pig

**DOI:** 10.1371/journal.pone.0146550

**Published:** 2016-01-11

**Authors:** Maëva Jégou, Florence Gondret, Annie Vincent, Christine Tréfeu, Hélène Gilbert, Isabelle Louveau

**Affiliations:** 1 INRA, UMR1348 Pegase, Saint-Gilles, France; 2 Agrocampus Ouest, UMR1348 Pegase, Rennes, France; 3 INRA, UMR1388 GenPhySE, Castanet-Tolosan, France; 4 Université de Toulouse, INP, ENSAT, UMR1388 GenPhySE, Castanet-Tolosan, France; 5 Université de Toulouse, INP, ENVT, UMR1388 GenPhySE, Toulouse, France; University of Lleida, SPAIN

## Abstract

The molecular mechanisms underlying feed efficiency need to be better understood to improve animal efficiency, a research priority to support a competitive and sustainable livestock production. This study was undertaken to determine whether pig blood transcriptome was affected by differences in feed efficiency and by ingested nutrients. Growing pigs from two lines divergently selected for residual feed intake (RFI) and fed isoproteic and isocaloric diets contrasted in energy source and nutrients were considered. Between 74 and 132 days of age, pigs (n = 12 by diet and by line) received a regular diet rich in cereals and low in fat (LF) or a diet where cereals where partially substituted by lipids and fibers (HF). At the end of the feeding trial, the total number of white blood cells was not affected by the line or by the diet, whereas the red blood cell number was higher (P<0.001) in low RFI than in high RFI pigs. Analysis of the whole blood transcriptome using a porcine microarray reveals a higher number of probes differentially expressed (DE) between RFI lines than between diets (2,154 versus 92 probes DE, P<0.01). This corresponds to 528 overexpressed genes and 477 underexpressed genes in low RFI pigs compared with high RFI pigs, respectively. Overexpressed genes were predominantly associated with translational elongation. Underexpressed genes were mainly involved in the immune response, regulation of inflammatory response, anti-apoptosis process, and cell organization. These findings suggest that selection for RFI has affected the immune status and defense mechanisms of pigs. Genes DE between diets were mainly related to the immune system and lipid metabolism. Altogether, this study demonstrates the usefulness of the blood transcriptome to identify the main biological processes affected by genetic selection and feeding strategies.

## Introduction

Production of efficient animals is an important issue for the livestock industry [1, 2] because this implies the reduction of the amount of feed resources needed to produce meat and contributes to reduce environmental wastes and emissions. Feed efficiency can be improved by breeding and feeding strategies. In recent years, residual feed intake (RFI) defined as the difference between the observed feed intake and the feed intake predicted from growth and maintenance requirements, has been studied as a measure of net feed efficiency in selection experiments [3]. Global performance differs between pigs selected for RFI, involving metabolism differences [4, 5] linked with gene expression variations in muscle [6]. The use of alternative feed resources (generally rich in fibers) that do not compete with food for humans is a challenge for the present and the future feeding strategies. However, interactions between RFI genotypes and these resources can affect body composition [7, 8]. An integrated approach combining genetics and nutrition is then necessary to get a better understanding of the complex biology underlying RFI and associated responses [9].

Peripheral blood is an accessible source of information. Indeed, the collection of blood samples is relatively easy compared to other tissues and does not alter the animal physiological status (anesthesia is unnecessary). It may further allow the investigation of kinetics of changes in different blood parameters. Advances in high-throughput technologies such as transcriptomics offer opportunities to answer complex biological questions. To date, genes expressed in peripheral blood cells have been shown to reflect physiological and pathological events occurring in different tissues [10, 11]. It has also been proposed that gene expression profiles from the whole blood or from peripheral blood mononuclear cells (PBMC) can highlight biological processes related to the regulation of body composition in human [12] and rodent [13]. Therefore, the transcriptome analysis of whole blood is a relevant approach to better understand the molecular mechanisms underlying differences in RFI, to determine the possible relationships between blood traits and production traits, and more generally to identify easily accessible targets to monitor physiological changes in response to factors such as nutrition. The current study was undertaken to determine whether diets with contrasted nutrients and genetic selection for RFI affect the whole blood transcriptome profiles of growing pigs.

## Materials and Methods

### Ethics statement

The care and use of pigs were performed in compliance with the European Union legislation (directive 2010/63/EU). The current protocol was approved by the local Ethics Committee in Animal Experiment of Rennes, France (Comité Rennais d'Ethique en matière d'Expérimentation Animale, CREEA, http://ethique.ipbs.fr/creeapresent.html; agreement N°07–2012). All animals were reared and killed in compliance with national regulations and according to procedures approved by the French veterinary Services at INRA Pegase facilities.

### Animals, diets and slaughtering

The pig lines and feeding trials used in the current study have been previously described in details [14]. Briefly, a total of 48 purebred French Large White castrated male pigs in the course of a divergent selection experiment for RFI (8^th^ generation of selection; n = 24 per line) were recruited. From 74 ± 3 days of age and an average body weight (BW) of 22.6 ± 0.5 kg, pigs were housed in full slatted-floor isolated pens. Within each line, they were randomly assigned to one of the two dietary groups (n = 12 per diet), and fed ad libitum either a low-fat, low-fiber (LF) diet or a high-fat, high-fiber (HF) diet. The two diets were mainly based on cereals (wheat and barley) and soybean meal. The HF diet was formulated by the partial replacing of cereals in the LF diet by wheat straw (11.5%) and a mixture of rapeseed and soya oils (7.5%). Each diet provided the same crude protein and metabolizable energy contents (Table 1). Growing and finishing formula were successively distributed to pigs during their growth (transition around 112 days of age). All pigs were killed at the same age (132.0 ± 0.5 days of age; average BW of 75.6 ± 1.1 kg). The killing procedure included electronarcosis and jugular exsanguination, and was performed 2 h after the first morning meal to obtain animals in a post-prandial state, in the INRA experimental slaughterhouse (Saint-Gilles, France).

**Table 1:** Composition of low-fat, low-fiber (LF) or high-fat, high-fiber (HF) diets given to pigs during the growing and finishing periods.

Composition^a^	Growing formula^b^		Finishing formula^b^	
	LF	HF	LF	HF
Fat	2.1	7.0	2.2	7.4
Starch	42.6	30.8	48.5	36.2
Crude protein	17.4	17.3	13.4	13.1
Neutral detergent fibers	12.8	17.7	11.5	18.3
Acid detergent fibers	3.8	8.5	3.5	7.9
Metabolizable energy	12.9	12.9	12.9	12.9

^a^Details on diet composition can be found in Gondret et al. [14]. Nutrient content is expressed in g per 100 g of feed (as fed basis). Metabolizable energy content is expressed in MJ per kg of feed.

^b^A growing formula was distributed to pigs during 6 weeks from 76 days of age, while the finishing formula was provided thereafter and until slaughter.

Initial blood samples were collected from the jugular vein at 74 days of age on living pigs.

Final blood samples were taken at exsanguination at the end of the feeding trial (132 days of age). Blood was collected into EDTA Venosafe tubes (Laboratoires Terumo, Guyancourt, France). For samples dedicated to RNA extraction, one volume of blood sample was mixed with one volume of lysis buffer from the Nucleospin 8 RNA blood kit (Macherey-Nagel, Lyon, France). The obtained mixture was then stored at -70°C for later analyses.

### Blood cell count

Whole blood cells can be separated into three categories. The first category refers to white blood cells (WBC), represented mainly by lymphocytes, monocytes and granulocytes. The two other categories refer to red blood cells (RBC) and platelet cells. Blood cell counts from those three categories were measured on whole blood samples taken at the end of the feeding trial using a hematology automatic cell counter calibrated for pigs (MS9-3, MELET SCHLOESING Laboratoires, Osny, France).

### RNA extraction

Total RNA were extracted from whole blood samples at both ages using a commercial kit (Nucleospin blood kit, Macherey Nagel, Hoerdt, France) according to the manufacturer’s instructions. Then, residual genomic DNA was removed from RNA samples by a DNase treatment (DNA-free kit, Applied Biosystems, Foster City, CA, USA) in the presence of a RNase inhibitor (Thermo Scientific, Illkirch, France). Extracted RNA samples were quantified using a NanoDrop ND-1000 spectrophotometer (Thermo Scientific, Illkirch, France). The integrity of isolated RNA was assessed using the Agilent RNA 6000 Nano kit with an Agilent 2100 Bioanalyzer (Agilent Technologies France, Massy, France). All samples met quality criteria. Ratios of A260/280 and A260/230 were greater than 1.8. Average RNA integrity number was of 9.2 with values ranging from 7.9 to 9.7.

### RNA labeling and microarray hybridization

Transcriptomics analyses were performed using a custom porcine microarray (8x60K, GPL16524, Agilent Technologies France, Massy, France) containing 60,306 porcine probes and derived at 71% from the porcine commercial Agilent-026440 microarray (V2, 44K, GPL15007), the remaining 29% correspond to a set of probes enriched with immune system, muscle and adipose tissue genes. Total RNA (100 ng) extracted from each whole blood samples taken at the end of the feeding trial was labelled individually with Cy3, using the One-Color Microarray-Based Gene Expression Analysis kit (Agilent Technologies) and following the manufacturer’s instructions. Briefly, fluorescent complementary RNA (cRNA) was generated by a two-step procedure using T7 RNA polymerase, which simultaneously amplified target and incorporated cyanine-labeled CTP. Samples were then purified with an RNeasy mini elute kit (Qiagen, Hilden, Germany). The hybridization reactions were performed for 17 h in Agilent’s SureHyb hybridization chambers containing 600 ng of Cy3-labeled cRNA per hybridization using Agilent’s Gene Expression Hybridization kit. Slides were disassembled, washed according to manufacturer’s instructions, and scanned at 3 μm/pixel resolution using the Agilent DNA Microarray Scanner G2505C, and images were analyzed with Agilent Feature Extraction Software (version 10.7.3.1) using the GE1_107_Sep09 extraction protocol. All microarray data have been deposited in NCBI’s Gene Expression Omnibus [15] and are accessible through GEO Subserie accession number GSE70838 (http://www.ncbi.nlm.nih.gov/geo/query/acc.cgi?acc=GSE70838).

### Microarray data analysis

All analyses were performed using the R software version 3.0.2 [16]. Raw spot intensities were first submitted to quality filtration based on four criteria: background intensity value, diameter, saturation and uniformity of the spot. Positives and negative controls probes were those available in the Agilent-026440 microarray. Intensities of filtered spots were log_2_ transformed and median-centered. Altogether, 37,113 spots were finally retained for statistical analyses. Data were submitted to an analysis of variance considering the fixed effects of line, diet and their interaction. Data were then submitted to Benjamini and Hochberg (BH) multiple testing correction procedure. For the diet effect, no genes were found to be differentially expressed (DE) after application of the BH correction. Therefore, DE probes with a fold change (FC) cutoff higher than |1.1| between compared groups, and an uncorrected P-value below 0.01 after statistical analysis [17] were then selected for further functional analysis.

The lists of DE probes were investigated by an enrichment analysis of specific Gene Ontology (GO) terms for Biological Processes (BP), using the functional annotation clustering of Database for Annotation, Visualization and Integrated Discovery (DAVID) bioinformatics resources (http://david.abcc.ncifcrf.gov) [18, 19]. Due to the low number of genes found to be DE between the two dietary treatments, the functional annotation clustering was performed only for genes DE between RFI lines. The list of DE genes in low RFI *versus* high RFI pigs was divided into two lists according to their over- or under-expression. The two lists were then uploaded using the corresponding official gene symbol, when applicable, and obtained with DAVID Gene Accession Conversion Tool. The GO terms_FAT were selected, to filter the broadest terms without overshadowing the more specific ones. The P-values for enrichment were computed by a modified Fisher’s exact test, using the *Homo sapiens* repository as background. The main GOBP term was examined with an enrichment score >1.3 and P≤ 0.1 after Benjamini Hochberg correction. For DE genes exhibiting the greatest overexpression or underexpression between RFI lines (FC > |2|) and for DE genes involved in the response to diets, a manual editing of GOBP terms was also performed using the QuickGo web-based tool [20].

### Quantitative Real-Time PCR (qPCR)

Expression of genes was further evaluated by qPCR. Final blood samples were used to validate transcriptomic results. Initial blood was used to investigate early expression of these genes. Complementary DNA was synthesized from 1 μg of total RNA, using a High Capacity cDNA Reverse Transcription kit (Applied Biosystems, Foster City, CA, USA). Primers (S1 Table) were designed from porcine sequences using Primer Express software 3.0 (Applied Biosystems). For each primer pair, the amplification efficiency of qPCR reaction was identified using calibration curves generated with seven decreasing concentrations of cDNA from pooled RNA blood samples (obtained from 5.18 to 1^E-3^ ng RNA). Amplification reactions were performed in duplicate in 12.5 μL with 1 ng of reverse-transcribed RNA and both forward and reverse primers (5 μM each) in 1X PCR buffer (Fast SYBR^®^ Green Master Mix, Applied Biosystems). A StepOnePlus Real Time PCR system (Applied Biosystems) was used. Thermal cycling conditions were as follows: 50°C for 2 min, 95°C for 20 s, followed by 40 cycles of denaturation at 95°C for 3 s, and annealing at 60°C for 30 s. Specificity of the amplification products was checked by dissociation curves analysis. This allows verifying that a single PCR product was produced. DNA topoisomerase 2-beta (*TOP2B*) and TATA-box-binding protein 1 (*TBP1*) genes were used as reference genes to calculate a normalization factor (NF) using geNorm algorithm [21]. For each gene, the normalized expression level N was calculated according to the formula developed by Pfaffl [22]:

$$N=E^{-\Delta Cq(sample-calibrator)}/NF$$

where E is calculated from the slope of calibration curve and Cq is the quantification cycle and calibrator is a pool of all blood samples. E was between 1.82 and 2.10 for all studied genes.

### Statistical analyses for blood cell counts

Analysis of variance was used to determine the effects of line, diet and their interaction with the R software (version 3.0.2). A P<0.05 was retained for statistical significance, and a P<0.1 were considered as a tendency.

## Results

### Performance and blood cell count traits

Phenotypic performances have been described in detail in our previous study [14] and are briefly summarized here. Irrespective of diet, the gain-to-feed ratio was higher in low RFI pigs than in high RFI pigs; the sum of the main fatty pieces in the carcass (backfat and belly) was lower while the sum of lean pieces was higher in low RFI pigs compared with high RFI pigs. Irrespective of line, no significant difference was elicited in gain-to-feed ratio between pigs fed the HF or LF diet. Pigs fed the HF diet had a reduced BW at slaughter and their carcass fat content was lower (around 26%) than in pigs fed the LF diet. At slaughter, the total number of white blood cells was similar (P>0.1) in the different groups of pigs (Table 2). The numbers of monocytes and neutrophil granulocytes (neutrophils) did not differ between groups. The number of lymphocytes was similar in low and high RFI pigs but tended (P<0.1) to be higher in pigs fed the HF diet than in pigs fed the LF diet. The number of red blood cells and associated parameters (hemoglobin and hematocrit) were markedly higher (P<0.001) in low RFI pigs than in high RFI pigs. These traits did not differ between diets, except a tendency for low RFI pigs fed the HF diet having a lower hematocrit than low RFI pigs fed the LF diet.

**Table 2:** Blood cell count in pigs with low or high residual feed intake (RFI) fed a low-fat, low-fiber (LF) or high-fat, high-fiber (HF) diet.

	Low RFI		High RFI		Effects	
Traits	LF	HF	LF	HF	Line	Diet
**WBC**^a^ **(x1000.mm**^**-3**^**)**						
Total WBC^a^	23.8 ± 1.2	24.9 ± 1.2	23.6 ± 0.7	25.7±1.0	NS	NS
Neutrophils	11.1 ± 0.8	10.6 ± 0.8	10.6 ± 0.4	12.0±0.8	NS	NS
Lymphocytes	12.1±0.6	13.7 ± 0.7	12.3 ± 0.5	13.1±0.6	NS	<0.1
Monocytes	0.56 ± 0.03	0.60 ± 0.04	0.63 ± 0.05	0.61±0.03	NS	NS
**RBC**^**b**^ **(x10**^**6**^**.mm**^**-3**^**) and associated parameters**						
Total RBC^b^	8.14 ± 0.16	7.96 ± 0.19	7.24 ± 0.14	7.38±0.16	<0.001	NS
Hemoglobin, g/dL	12.8 ± 0.3	12.0 ± 0.3	11.4 ± 0.3	11.4±0.2	<0.001	NS
Hematocrit, %	47.1 ± 1.0	44.5 ± 1.1	42.8 ± 0.7	42.4±0.5	<0.001	<0.1

^a^WBC: white blood cell count.

^b^RBC: red blood cell count.

Values are means ± SEM (n = 12 pigs per diet and per line). P-value obtained from analysis of variance for the effects of line and diet. There was no significant line × diet interaction (P > 0.1). NS, not significant (P > 0.1).

### RFI and diet effects on whole blood transcriptome

In whole blood taken at the end of the feeding trial, 2,075 probes corresponding to 1,005 genes were declared as DE (P < 0.01) between RFI lines and 82 probes corresponding to 45 genes were DE between pigs fed the HF and LF diets (Table 3). An interaction between line and diet (P < 0.01) was observed for 106 probes corresponding to 74 annotated genes. The detailed list of these corresponding genes can be found in supplementary file S2 Table.

**Table 3:** Number of genes differentially expressed in blood of pigs divergently selected for low or high residual feed intake (RFI) fed a low-fat, low-fiber (LF) or high-fat, high-fiber (HF) diet.

	Low RFI / High RFI^a^		HF / LF^b^	
DE genes^c^	Overexpressed	Underexpressed	Overexpressed	Underexpressed
Total number	528	477	39	6
FC^d^ ≥ 1.5	44	65	8	-
FC^d^ ≥ 2	10	17	2	-

^a^Low RFI versus high RFI ratio, overexpressed and underexpressed in low RFI compared with high RFI pigs.

^b^HF versus LF ratio, overexpressed and underexpressed in pigs fed the HF diet compared with pigs fed the LF diet.

^c^DE: Differentially expressed; P < 0.01.

^d^FC: Fold change ratio.

#### RFI effects on whole blood transcriptome

Among the 2,075 DE probes (P < 0.01; adjusted P value ≤ 0.1 for 88% of the probes and ≤ 0.17 for the other probes) in response to RFI selection, 982 probes corresponding to 528 annotated unique genes were overexpressed whereas 1093 probes corresponding to 477 annotated genes were underexpressed in low RFI pigs compared with high RFI pigs. A large proportion of genes (83 to 90%) exhibited fold changes in expression below |1.5| between the two lines (Table 3). Functional analysis revealed an overrepresentation of genes related to translational elongation among the overexpressed genes in the low RFI pigs compared with high RFI pigs (Table 4). Conversely, genes that were underexpressed in low RFI pigs compared with high RFI pigs shared GO terms associated to 8 different pathways: defense response, leukocyte activation, regulation of inflammatory response, negative regulation of molecular function, antigen processing and presentation, anti-apoptosis, positive regulation of immune system process and regulation of cell adhesion (Table 4).

**Table 4:** Relevant GO biological processes in blood as affected by selection for RFI.

GO term^a^		Genes^b^
**Overexpressed genes in low RFI line compared with high RFI line**		
GO:0006414	Translational elongation	*RPSA*, *EEF1A1*, *RPL14*, *RPL15*, *RPL35*, *RPL23A*, *SELT*, *RPS3*, *RPS25*, *RPS18*, *RPL6*, *RPS13*, *RPL10*, *RPL10A*, *RPS21*, *UBA52*, *RPS23*
**Underexpressed genes in low RFI line compared with high RFI line**		
GO:0006952	Defense response	*KYNU*, *NMI*, *FGR*, *LY86*, *CCR1*, *CLU*, *TLR1*, *TLR2*, *CXCR2*, *PRDX1*, *IL10*, *IL10RB*, *BCL2*, *TAP1*, *CSF3R*, *NFATC4*, *CD24*, *THBS1*, *BLNK*, *F12*, *TLR10*, *PTGER3*, *LY96*, *BECN1*, *HCK*, *IL1RN*, *HLA-C*, *C4BPB*, *HLA-B*, *CD40*, *C4BPA*, *STAT3*, *CD84*, *CD83*, *CD55*, *CD19*, *HIST2H2BE*, *CLEC7A*, *CLEC5A*, *CD14*
GO:0045321	Leukocyte activation	*ZBTB32*, *BST2*, *TLR1*, *TLR2*, *CXCR2*, *CD40*, *TPD52*, *SKAP2*, *IL10*, *CBLB*, *PKNOX1*, *PSEN1*, *ULBP1*, *BCL2*, *BCL11A*, *MS4A1*, *ADAM17*, *BCL6*, *CLEC7A*, *CD24*, *SYK*, *BLNK*, *ADAM9*
GO:0050727	Regulation of inflammatory response	*F12*, *ADRB2*, *PTGS2*, *SERPINF1*, *TGM2*, *CMA1*, *BCL6*, *JAK2*, *CD24*, *IL10*
GO:0044092	Negative regulation of molecular function	*NF1*, *PKIG*, *NPR3*, *PKIA*, *PROX1*, *PDCD4*, *PSMB8*, *PSMB9*, *SH3BP5*, *SPRY2*, *ADRB2*, *PSEN1*, *PSME2*, *CDKN2D*, *RGS4*, *PSMA3*, *UBC*, *SORT1*, *JAK2*, *UBE2D1*, *BUB3*, *DHCR24*
GO:0019882	Antigen processing and presentation	*HLA-H*, *ULBP1*, *HLA-DRB3*, *HLA-A*, *ERAP1*, *HLA-C*, *CD1A*, *HLA-B*, *HLA-DOB*, *PSMB8*, *PSMB9*
GO:0006916	Anti-apoptosis	*BECN1*, *SPHK1*, *CLU*, *BIRC3*, *ANXA4*, *IL10*, *TNFSF13B*, *PSEN1*, *BCL2*, *CDKN2D*, *TGM2*, *UBC*, *ADAM17*, *THBS1*, *DHCR24*
GO:0002684	Positive regulation of immune system process	*CLU*, *TLR2*, *C4BPB*, *CD40*, *C4BPA*, *CD83*, *CBLB*, *CD55*, *CD19*, *TNFSF13B*, *PSEN1*, *ADAM17*, *BCL6*, *CD24*, *CLEC7A*, *THBS1*, *SYK*
GO:0030155	Regulation of cell adhesion	*VAV3*, *GSN*, *BCL2*, *NF1*, *CCDC80*, *TGM2*, *BCL6*, *JAK2*, *CD24*, *THBS1*, *TPM1*, *ADAM9*

^a^Gene ontology (GO) identification number and term of the biological process. Benjamini-Hochberg adjusted P-value varied from 0.1 and 8.10^−05^.

^b^Unique genes included in each pathway.

Differentially expressed genes exhibiting the greatest overexpression or underexpression (FC > |2|) in low RFI pigs compared with high RFI lines (Table 5) were also considered to extend this global functional analysis. Among these eight genes having the greatest overexpression in the low *versus* high RFI lines, the majority had a documented role in the immune system (*IFITM1*, *SLPI*, *IL6ST*, and *TRAF6*); the other were mainly related to DNA organization (*NPM2*), translation (*EIF1B*), cellular homeostasis (*SLCO2B1*) and peptidase regulation (*WFDC2*). With respect to genes exhibiting the greatest underexpression in low RFI pigs compared with high RFI pigs, their roles were also associated with immune system (*NMI*, *CHIT1*, *SLA-DOA*), oxidative process (*GPX3*, *CAPNS1*, *OAZ3*), DNA organization (*HMG20A*), angiogenesis and the control of blood volume (*SERPINF1*, *HTRA1*, *NPR3*).

**Table 5:** Top-ranked genes with fold changes in expression greater than |2| identified in the whole blood of pigs divergently selected for low or high residual feed intake (RFI).

Gene symbol	Main biological process^a^	FC^b^	P-value^c^
**Overexpressed genes in low RFI line compared with high RFI line**			
*IFITM1*	GO:0009607 response to biotic stimulus	4.34	2.6.10^−04^
*NPM2*	GO:0006338 chromatin remodeling	3.11	3.5.10^−04^
*EIF1B*	GO:0006412 translation	2.51	4.8.10^−13^
*SLPI*	GO:0045071 negative regulation of viral genome replication	2.33	6.1.10^−05^
*IL6ST*	GO:0019221 cytokine-mediated signaling pathway	2.20	1.3.10^−03^
*WFDC2*	GO:0010466 negative regulation of peptidase activity	2.16	4.5.10^−08^
*TRAF6*	GO:0002726 positive regulation of T cell cytokine production	2.08	1.4.10^−06^
*SLCO2B1*	GO:0006811 ion transport	2.04	6.9.10^−06^
**Underexpressed genes in low RFI line compared with high RFI line**			
*GPX3*	GO:0006979 response to oxidative stress	-6.07	1.3.10^−06^
*HMG20A*	GO:0006338 chromatin remodeling	-4.69	1.2.10^−08^
*CAPNS1*	GO:0006508 proteolysis	-4.09	3.1.10^−07^
*OAZ3*	GO:0043086 negative regulation of catalytic activity	-4.00	1.1.10^−06^
*NMI*	GO:0045824 negative regulation of innate immune response	-3.49	1.6.10^−07^
*SERPINF1*	GO:0016525 negative regulation of angiogenesis	-3.28	3.8.10^−08^
*HTRA1*	GO:0030512 negative regulation of transforming growth factor beta receptor signaling pathway	-2.68	1.1.10^−09^
*NPR3*	GO:0008217 regulation of blood pressure	-2.17	1.4.10^−07^
*CHIT1*	GO:0006030 chitin metabolic process	-2.12	4.6.10^−07^
*SLA-DOA*	GO:0019882 antigen processing and presentation	-2.12	1.9.10^−11^

^a^The gene ontology (GO) term for biological process was manually obtained from the QuickGO web-based tool for each gene name.

^b^Fold change value is expressed as the expression ratio of low RFI pigs *versus* high RFI pigs. Ratio was inversed and preceded by a minus sign for value less than 1 (i.e., a ratio of 0.5 is expressed as -2).

^c^P-value obtained from analysis of variance for the effect of line. The highest P-value is reported when several probes are differentially expressed for a unique gene. Adjusted P-value varied from 0.05 and 1.10^−09^.

#### Diet effects on whole blood transcriptome

The number of DE probes in response to diets was markedly lower than that observed in response to genetic selection (Table 3). Among the 82 DE probes between diets, 62 probes corresponding to 39 annotated genes were overexpressed, whereas 20 probes corresponding to 6 annotated genes were underexpressed in pigs fed the HF diet compared with pigs fed the LF diet. The majority of these genes (78%) had fold change in expression between the two diets below |1.5| (Table 3). As indicated in Table 6, among the 16 genes overexpressed in pigs fed the HF diet compared with pigs fed the LF diet, six genes were related to the immune system (*PR39*, *PMAP-23*, *CD5L*, *TCN1*, *PGLYRP1* and *HP*), three genes were involved in cellular metabolism such as collagen fiber assembly or cytoskeletal arrangement (*BGN*, *PLEK2*, *RASL11B*), two genes were involved in lipid storage (*CPT1A*, *LCN2*), two genes had a role in erythropoietic system (*EPOR*, *KLF1*), two genes had a role in DNA synthesis (*NOCL4*; *REXO2*) and one gene was involved in embryogenesis (*SHISA2*). Conversely, three genes were underexpressed in pigs fed the HF diet and were involved in lipid metabolism (*PSAP*), RNA localization (*RAE1*), and nucleotides phosphorylation (*NTPCR*).

**Table 6:** Differentially expressed genes in pigs fed diets with contrasted energy source and nutrients.

Gene symbol	Main biological process^a^	FC^b^	P-value^c^
**Overexpressed genes in HF pigs compared with LF pigs**			
*PR39*	GO:0042742 defense response to bacterium	3.06	6.0.10^−03^
*PMAP-23*	GO:0042742 defense response to bacterium	2.84	7.3.10^−03^
*CD5L*	GO:0006898 receptor-mediated endocytosis	1.72	3.3.10^−03^
*LCN2*	GO:0006810 transport	1.61	8.4.10^−03^
*TCN1*	GO:0015889 cobalamin transport	1.60	9.2.10^−03^
*BGN*	GO:0019800 peptide cross-linking via chondroitin 4-sulfate glycosaminoglycan	1.53	9.0.10^−03^
*PGLYRP1*	GO:0050728 negative regulation of inflammatory response	1.51	8.1.10^−03^
*EPOR*	GO:0038162 erythropoietin-mediated signaling pathway	1.46	5.5.10^−03^
*HP*	GO:0006508 proteolysis	1.46	9.7.10^−03^
*PLEK2*	GO:0035556 intracellular signal transduction	1.33	6.6.10^−03^
*RASL11B*	GO:0007264 small GTPase mediated signal transduction	1.29	1.1.10^−04^
*SHISA2*	GO:0007275 multicellular organismal development	1.28	8.3.10^−04^
*NOC4L*	GO:0042254 ribosome biogenesis	1.27	1.0.10^−03^
*KLF1*	GO:0030218 erythrocyte differentiation	1.25	6.4.10^−03^
*CPT1A*	GO:0032000 positive regulation of fatty acid β-oxidation	1.24	1.8.10^−03^
*REXO2*	GO:0090305 nucleic acid phosphodiester bond hydrolysis	1.21	9.4.10^−03^
**Underexpressed genes in HF pigs compared with LF pigs**			
*NTPCR*	GO:0016311 dephosphorylation	-1.29	4.9.10^−03^
*RAE1*	GO:0071407 cellular response to organic cyclic compound	-1.24	5.2.10^−03^
*PSAP*	GO:0006629 lipid metabolic process	-1.20	7.3.10^−03^

^a^The gene ontology (GO) term for biological process (BP) was manually obtained from the QuickGO web-based tool for each gene name.

^b^Fold change (FC) value is expressed as the expression ratio of pigs fed the HF (high fiber, high fat) diet versus pigs fed the LF (low fat low fiber) diet. FC is inversed and preceded by a minus sign for value less than 1 (i.e. a ratio of 0.5 is expressed as -2).

^c^P-value obtained from analysis of variance for the effect of diet. The highest P-value is reported when several probes are differentially expressed for a unique gene.

### Data validation by qPCR analysis

**Fig 1:**
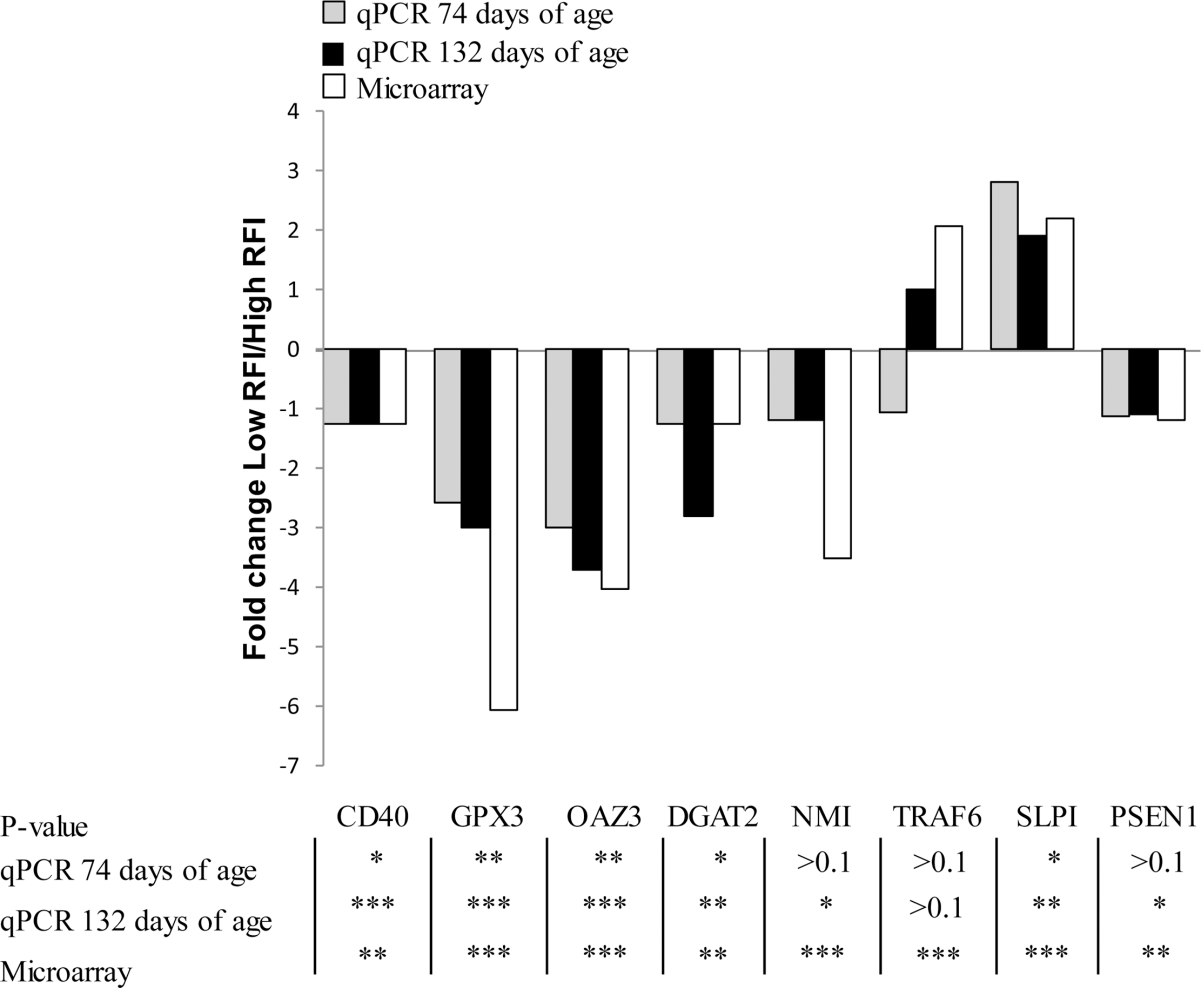
Comparison of microarray and qPCR data for eight genes in pigs divergently selected for low or high residual feed intake (RFI). Transcriptomic differences between the two lines were validated by qPCR at 132 and 74 days of age (onset of the growing period). For values related to microarray, the highest P-value is reported when several probes are differentially expressed for a unique gene (*P < 0.05; **P < 0.01; ***P<0.001). Fold change value is expressed as the expression ratio of low RFI to high RFI samples; ratio was inversed and preceded by a minus sign for value less than 1 (i.e., a ratio of 0.5 is expressed as -2). *CD40*, tumor necrosis factor receptor superfamily member 5; *GPX3*, glutathione peroxidase 3; *OAZ3*, ornithine decarboxylase antizyme 3; *DGAT2*, diacylglycerol O-acyltransferase 2; *NMI*, N-myc interactor; *TRAF6*, TNF receptor-associated factor 6; *SLPI*, secretory leukocyte peptidase inhibitor; *PSEN1*, presenilin 1.

The expression of eight genes identified as DE between RFI lines by transcriptome analysis was checked by qPCR (Fig 1). With the exception of *TRAF6* (P = 0.16), the range of difference (P < 0.05) in the expression of *CD40*, *GPX3*, *OAZ3*, *DGAT2*, *NMI*, *PSEN1* and *SLPI* genes was consistent with microarray analyses. Expressions of those eight genes were also determined in 74-day-old pigs (Fig 1). The differences in expression levels between RFI lines observed at 132 days of age were detected at the onset of the growing period for *CD40*, *GPX3*, *OAZ3*, *DGAT2* and *SLPI* genes (P < 0.05). For three other genes (*NMI*, *TRAF6*, *PSEN1*), there was no difference (P > 0.1) between low RFI and high RFI pigs at this early stage of growth.

**Fig 2:**
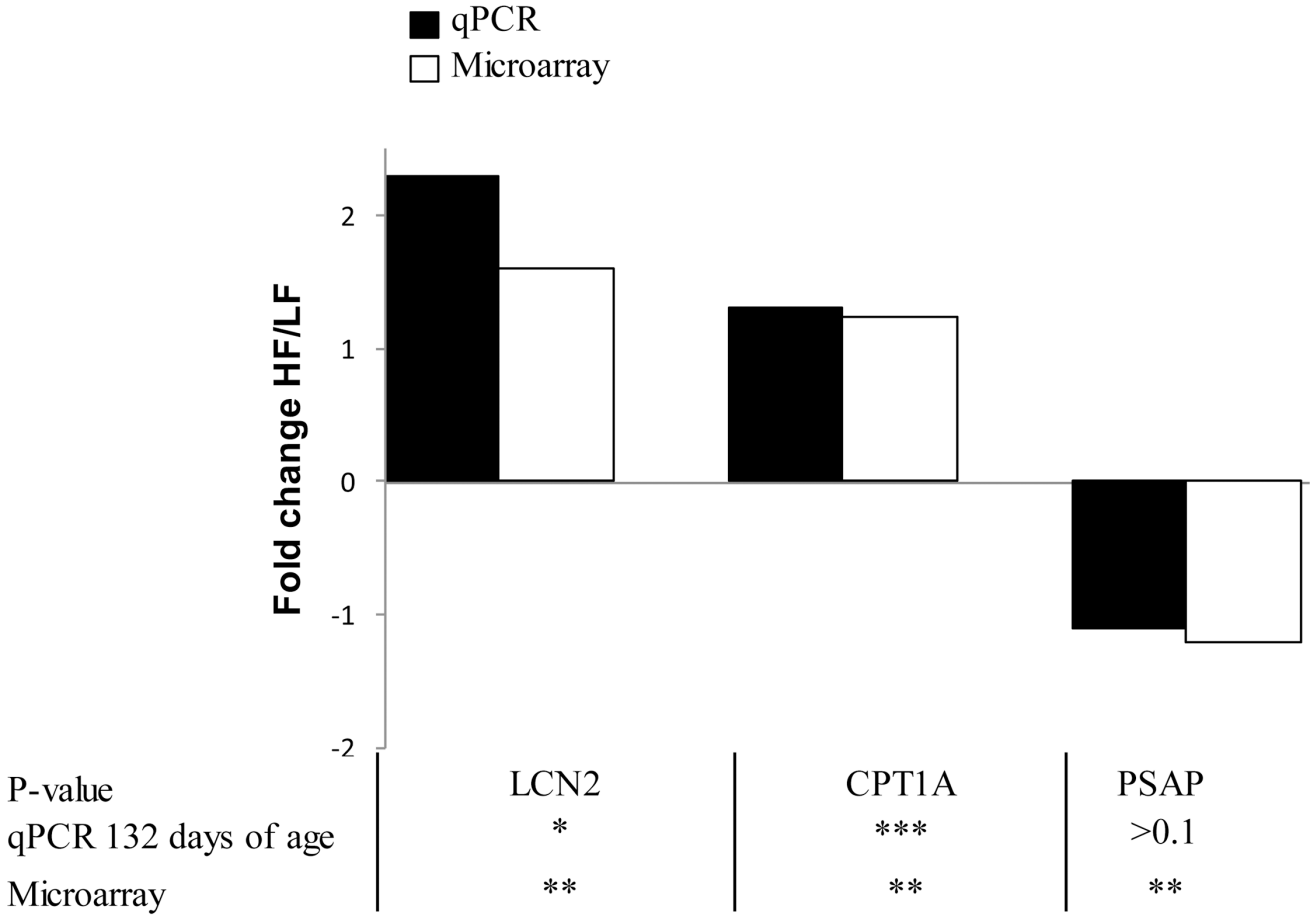
Comparison of microarray and qPCR data for three genes in pigs fed the HF or LF diet. For values related to microarray, the highest P-value is reported when several probes are differentially expressed for a unique gene (*P < 0.05; **P < 0.01; ***P < 0.001). Fold change value is expressed as the expression ratio of HF (high fiber high fat, n = 24) to LF (low fiber low fat, n = 24) diets; ratio was inversed and preceded by a minus sign for value less than 1 (i.e., a ratio of 0.5 is expressed as -2). *LCN2*, lipocalin 2; *CPT1A*, Carnitine palmitoyltransferase 1A; *PSAP*, prosaposin.

Finally, the expression levels of three genes (*LCN2*, *CPT1A*, *PSAP*) identified as DE between pigs fed the two diets by transcriptome analysis were also determined by qPCR at 132 days of age (end of the feeding trial), and the range of difference was consistent (P < 0.001) with microarray analyses for *LCN2* and *CPT1A*, while *PSAP* expression by qPCR did not significantly differ (P > 0.10) between HF and LF pigs (Fig 2).

## Discussion

The current study provides new evidence that the investigation of the expression of genes in whole blood allowed clear identification of biological pathways and indicator traits involved in the response to genetics and nutrition strategies. To date, the majority of studies including a microarray analysis from whole blood or PBMC have been performed in adults exhibiting several disorders [23]. Therefore, the current study provides novel information on the response of blood transcriptome during growth. This study further supports the interest to use RNA extracted from whole blood which is easier to obtain compared with RNA extracted from PBMC which requires supplementary steps for cell isolation. In the current study, there was no significant difference in the numbers of lymphocytes, monocytes and neutrophils between the experimental groups, so that identified DE genes after microarray analysis were not related to differences in the number of WBC. These observations differ from a recent study reporting a lower number of WBC, especially lymphocytes and monocytes, in Yorkshire pigs selected for low RFI compared with pigs selected for high RFI and considered at younger age (35–42 days) [24]. With respect to RBC, their total number was higher in low RFI than in high RFI pigs in the current study, while this number did not differ between the divergent RFI lines in the study of Mpetile et al. [24]. It is important to note that other parameters related to RBC were similarly affected by RFI selection in both studies, with higher hemoglobin plasma concentration and higher hematocrit percentage in low RFI pigs than in high RFI pigs. The current findings may reflect differences in intensity of selection, age of pigs considered for the measurement of blood cell traits or environmental stimuli during pig housing.

The current study clearly indicates that genetics has a greater impact on blood gene expression profiles than dietary treatment. In a rat model, genetic background has been shown to have a much larger impact on PBMC transcriptome than on heart transcriptome [25]. With the current findings, it can be hypothesized that the identified DE genes in whole blood may reflect changes that occur in a similar manner in several tissues of the organism in response to genetic selection for RFI. Indeed, it has been shown that the expression of a large number of genes was shared among all tissues including blood cells [10].

The most important pathways shared by genes DE between RFI lines in the blood concern immunity and defense mechanisms. Most of these genes were underexpressed in low RFI pigs compared with high RFI pigs, although some of them (*IFITM1*, *SLPI*, and *IL6ST*) were rather overexpressed in low RFI pigs. For instance, the *CD40* gene coding for a TNF receptor superfamily member required for the B-cell function was found to exhibit a lower expression in the low RFI pigs compared with the high RFI pigs at both examined ages. The expression level of *PSEN1*, a gene participating to T cell activation, was decreased only in 132-day-old pigs. Conversely, the *SLPI* gene, coding for an antimicrobial protein having inhibitory effects that contribute to the immune response by protecting epithelial surfaces, was overexpressed in low RFI pigs at both ages. An overexpression of the *IL6ST* gene was also observed in low RFI pigs, a gene encoding a signal transducer shared by many cytokines including interleukin 6. Finding many genes related to immunity in the whole blood transcriptome is expected considering the fact that blood cells constitute one of the first lines of immune defense [10]. A recent study in pigs showing that peripheral blood transcriptome is a relevant source to identify genes related to the immune function responses and to predict the efficiency of individual’s immune response further supports our data [26]. From the current data, it is however not possible to predict this efficiency of immune response in RFI pigs. It has been suggested that livestock animals selected for high production efficiency traits may be more vulnerable to diseases and stressors [27, 28]. Nevertheless, the few available data dealing with the inflammatory response of the two RFI lines do not allow a clear conclusion [29–30]. A lower level of basal inflammation, as indicated by reduced concentration of the acute phase protein haptoglogin, has been recently reported in low RFI pigs compared with high RFI pigs [29]. In response to an immune challenge induced by infection with the porcine reproductive and respiratory syndrome virus [31], pigs selected for a low RFI compared with high RFI pigs had a greater immune response and their growth rate was less affected. Further studies are thus needed to confirm the higher capacity of low RFI pigs and to get a better understanding of the immune capacity and inflammatory response of the two RFI lines.

Genes associated with translational elongation were overexpressed in low RFI pigs compared with high RFI pigs, including *eiF1B* gene involved in translational initiation as one of the top overexpressed genes in the former pigs. Detectable expression of *eiF1B* gene has been previously demonstrated after microarray analyses in normal whole blood from Human patients [32]. An increase in the expression of genes encoding initiation and elongation factor subunits has been also recently reported in skeletal muscle of low RFI pigs of the sixth and seventh generations of the same selection experiment [6]. These findings may reflect a greater protein synthesis in low RFI pigs than in high RFI pigs, to support increased muscle gain in the former pigs during the growing period [5]. This remains to be further investigated. Indeed, translation initiation signaling proteins did not differ in skeletal muscle between low and high RFI lines [4, 33].

The expression of genes encoding gluthatione peroxidase 3 (*GPX3*) and ornithine decarboxylase antizyme 3 (*OAZ3*) was also found as DE in low *versus* high RFI pigs at both ages. The same genes have been previously reported to be DE in skeletal muscle between pigs of the former generations of selection [6]. Those two genes have a role in oxidative metabolism. *GPX3*, encoding an enzyme involved in the detoxification of hydrogen peroxide when reactive oxygen species were produced, accounts for the major antioxidant activity in the plasma. *OAZ3*, encoding an enzyme which is an inhibitor of ornithine decarboxylase, itself involved in the anti-oxidant defense, converts ornithine in putrescine, the first step in the synthesis of polyamines that have antioxidant properties. These findings argue for a lower oxidative stress in low RFI pigs [34] and the expression of these genes may be considered as relevant indicators of RFI status in the pig.

Only few genes were found DE in whole blood samples collected from pigs fed the HF or LF diets, despite a large impact of the dietary treatment on growth and body composition of pigs from the two RFI lines [14, 35]. Between normal weight and diet-induced obese (cafeteria diet) rats, exhibiting large differences in body weight and adiposity index, the number of genes affected by the diet in PBMC (566 genes; [36]) was much higher than in the present study. The difference in the extent of the gene response between our study and the above-mentioned ones are unknown. This may be related to differences in nutrient composition or energy level of the diet. Alternatively, the difference may be related to the fact that an increase in adipose tissue mass occurred in those studies, whereas there was a decrease in the fat mass of pigs fed the HF diet compared with pigs fed the LF diet.

Despite the low number of genes DE in response to the HF compared with the LF diet, the current study highlighted differences in the expression of genes related to the immune system and lipid metabolism as shown reported in PBMC of miniature pigs fed a hyperlipidic diet *versus* hyperlipidic and hyperglucidic diet for 27 weeks [37]. Overexpressed genes in pigs fed the HF diet were mainly involved in host defense mechanism (*PR39*, *PMAP-23*, *CD5L*, *PGLYRP1*). With respect to lipid metabolism, two genes were also identified as DE in whole blood of pigs fed the two diets. The *CPT1A* (carnitine palmitoyl transferase 1A) gene, encoding an enzyme which is localized in the inner membrane of the mitochondria and participates to the entrance of fatty acids in the mitochondria for β-oxidation, was overexpressed in HF pigs compared with LF pigs. This may reflect a higher fatty acid oxidative capacity in blood cells of HF pigs, as observed in rats [38]. Other studies have shown that *CPT1A* gene expression in PBMC did not vary between overweight and control rats [39], or between obese and normal weight subjects in human [40]. Conversely, the expression of *CPT1A* was higher in overweight children than in normal weight children [41]. In our study, the fact that HF pigs were leaner and lighter than LF pigs [14] suggests no direct relationships between *CPT1A* expression in peripheral blood and changes in adipose tissue mass.

The gene encoding lipocalin 2 (LCN2) was also overexpressed in pigs fed the HF diet compared with pigs fed the LF diet. Lipocalin 2 also named neutrophil gelatinase-associated lipocalin (NGAL) has been recently characterized as an adipokine playing putative roles in glucose and lipid metabolism [42, 43]. This adipokine shares structural similarities with fatty acid binding proteins and retinol-binding proteins. Recent studies have shown a higher LCN2 expression level in PBMC of adults exhibiting higher adiposity [44] and (or) higher body weight and body mass index [45]. With this contrasted findings, further studies are needed to determine whether there is a possible relationship between peripheral blood LCN2 expression and adipose tissue mass variation.

In summary, the current study based on the investigation of pigs selected for feed efficiency and fed two diets contrasted in energy sources indicate that transcriptomics analyses is an additional tool to study variations of phenotypes in a dynamic way throughout the life of the animal. It also supports the potential use of blood transcriptome to highlight biomarkers for future selection process in pig or in other species.

## Supporting Information

S1 Table Primer sequences used for analysis of gene expression by qPCR. (DOCX)

S2 Table Probes expressed in whole blood of pigs selected for low or high residual feed intake (RFI) fed a low-fat, low-fiber (LF) or high-fat, high-fiber (HF) diet, with significant interaction between line and diet. (XLSX)
