# Supplementary material for: Whole Blood Transcriptomics Is Relevant to Identify Molecular Changes in Response to Genetic Selection for Feed Efficiency and Nutritional Status in the Pig
Source: PLoS One. 2016 Jan 11;11(1):e0146550. doi: 10.1371/journal.pone.0146550 (PMC4709134; doi:10.1371/journal.pone.0146550)
Supplement: S1 Table — (DOCX) [file pone.0146550.s001.docx]

**S1 Table. Primer sequences used for analysis of gene expression by qPCR.**

| **Gene symbol** | **Description** | **Accession number^a^** | **Primer sequence (5’-3’)^b^** |
| --- | --- | --- | --- |
| CD40 | CD40 molecule, tumor necrosis factor receptor superfamily member 5 | NM_214194 | F : GCCCGCCAGGACAGAAA  R : GGAACTGCAAGGAAGGCATTC |
| CPT1A | Carnitine palmitoyltransferase 1A | XM_005660602 | F: TTAAAGTCCTGGTGGGCTACAA  R: CCTCGCCGCCTGAATGT |
| DGAT2 | Diacylglycerol O-acyltransferase 2 | HQ403606 | F: CCTGATGTCTGGAGGCATCTG  R: CACGATGATGATGGCATTGC |
| GPX3 | Glutathione peroxidase 3 | NM_001115155 | F : GCTTCCCCTGCAACCAATT  R : GGACATACCTGAGAGTGGACAGAA |
| LCN2 | Lipocalin 2 | XM_005660462 | F: TCGCAATCGACCAGTGCAT  R: TGGGCAAAGGCTGAAGACAT |
| NMI | N-myc (and STAT) interactor | XM_005652427 | F: GTGGAGAGCGTGGAGTATGACA  R: TCAGCAACTCCACTTTCCACAA |
| OAZ3 | Ornithine decarboxylase antizyme 3 | NM_001301406 | F : ATTGTATTCGGCTGGGAACCT  R : GGCGGAAGTGGAAGTCTAGCT |
| PSAP | Prosaposin | XM_005671043 | F : ACAAGCCAACCGTGAAATCC  R : CAGTGGCATTGTCTTTCAACATG |
| PSEN1 | Presenilin 1 | XM_005666340 | F : GTCACGATCTGCTGTACAGGATCT  R : ATCCAAGTTTTACTCCCCTTTCTTC |
| SLPI | Secretory leukocyte peptidase inhibitor | NM_213870 | F : TGCTATCACGAACCCAGTTAAGG  R : GAGCATCATACACTGGCCATAGAC |
| TRAF6 | TNF receptor-associated factor 6 | XM_005652801 | F: CCCCCTGTGGTCATAGGTTCT  R: GTGACCTGCATCCCTTATTGACT |
| TBP1^c^ | Telomeric DNA binding protein 1 | XM_005658569 | F : AACAGTTCAGTAGTTATGAGCCAGA  R: AGATGTTCTCAAACGCTTCG |
| TOP2B^c^ | Topoisomerase (DNA) II beta | AF222921 | F: AACTGGATGATGCTAATGATGCT  R: TGGAAAAACTCCGTATCTGTCTC |

^a^Accession number in the National Center for Biotechnology Information (NCBI) or Ensembl project database for pig sequences. F and R indicated respectively.

^b^F: forward primers; R: reverse primers.

^c^Gene used as reference for qPCR normalization.
